# Stuck Prosthetic Valves: Clinical Implications of Pannus Formation and Gradient Measurement in Surgical Outcomes

**DOI:** 10.3390/jcm14020515

**Published:** 2025-01-15

**Authors:** Muhammet Fethi Sağlam, Emrah Uguz, Kemal Eşref Erdogan, Hüseyin Ünsal Erçelik, Murat Yücel, Mete Hıdıroglu, Erol Şener

**Affiliations:** 1 Department of Cardiovascular Surgery, Ankara Yıldırım Beyazıt University Faculty of Medicine, 06010 Ankara, Türkiye; emrahuguz@gmail.com (E.U.); kemal_esref@hotmail.com (K.E.E.); metetaha@hotmail.com (M.H.); drerolsener@gmail.com (E.Ş.); 2 Ankara Bilkent City Hospital, 06800 Ankara, Türkiye; unsalercelik@gmail.com (H.Ü.E.); dryucelmurat@gmail.com (M.Y.)

**Keywords:** stuck prosthetic valve, pannus formation, left ventricular ejection fraction, gradient measurements, valve replacement

## Abstract

**Objective:** Stuck prosthetic valves, often resulting from pannus formation or thrombus accumulation, represent a critical complication in prosthetic valve management, carrying significant risks for morbidity and mortality. This study aims to identify factors associated with stuck valve development and assess the effectiveness of interventions in restoring normal valve function. **Methods:** A total of 27 patients with stuck valves were analyzed, including mitral, aortic, and tricuspid valve cases. Metallic valves were initially implanted in all patients. Interventions included pannus cleaning in suitable cases and valve replacement when necessary, with the replacement being either metallic and biological based on clinical indications. Preoperative and postoperative ECG rhythms, left ventricular ejection fraction (LVEF) values, and gradient measurements were evaluated across patient groups. **Results:** No significant difference was found in time since initial surgery across valve types (*p* = 0.67), except in mitral valves, where time was longer in the replacement group (*p* = 0.02). Maximum gradients were higher in the pannus cleaning group for mitral valves (*p* = 0.03), while overall gradient values showed no significant differences. Postoperative left ventricular ejection fraction improved significantly in all groups (*p* < 0.001). **Conclusions:** The findings highlight the importance of timely intervention in managing stuck prosthetic valves, which are associated with severe hemodynamic compromise and embolic risk. Pannus cleaning emerged as a viable alternative in selected cases where the obstruction was localized, with the valve structure otherwise intact. Biological valve replacements demonstrated superior rhythm stabilization in this study, although definitive conclusions are constrained by the minimal sample size (*n* = 2). Future research should focus on expanding sample sizes and incorporating comprehensive preoperative analyses to better inform surgical and clinical management strategies.

## 1. Introduction

Cardiovascular diseases remain a major global health issue, posing significant threats to public health and being one of the leading causes of morbidity and mortality [1]. Heart valve diseases represent a critical subset of cardiovascular disorders, especially affecting the quality of life and increasing mortality rates in older adults. In the management of heart valve diseases, valve replacement or repair often becomes a necessary intervention. Surgically implanted mechanical and bioprosthetic valves play a vital role in maintaining hemodynamic stability and improving long-term survival rates for these patients [2,3].

However, with the increasing clinical use of prosthetic valves, structural and functional deterioration in these valves over time has emerged as a serious concern. Among the complications associated with prosthetic valves, “stuck valve” syndrome is a mechanical dysfunction that directly impacts valve function [4]. A stuck valve is often caused by thrombus formation or pannus growth, both of which restrict the valve’s ability to open and close properly. This condition has substantial risks for both patients and clinicians, as an impaired valve function leads to severe hemodynamic changes and can ultimately result in life-threatening situations [5,6,7].

Pannus formation, a common complication in mechanical valves, restricts prosthetic valve function by growing within the valve structure itself, directly limiting the movement of the valve leaflets. Composed of fibrotic tissue resulting from an inflammatory response, the pannus obstructs leaflet motion, leading to a “stuck” valve. While this complication typically arises over time, it can also occur in the early postoperative period, particularly if the patient is not compliant with regular warfarin use or does not maintain consistent blood coagulation monitoring. Stuck valve complications significantly impact patient quality of life and often necessitate additional interventions to restore valve function [8,9].

In this study, we aimed to investigate the role of pannus formation in the development of stuck valves and its impact on clinical outcomes. Specifically, we examined the effects of the time elapsed since the initial surgery, the maximum and mean gradient measurements of the valve, and pannus formation on the impact of stuck valve development. Additionally, changes in preoperative and postoperative electrocardiogram (ECG) rhythms and left ventricular ejection fraction (LVEF) values were analyzed. Our findings are expected to contribute to clinical decision-making for the long-term follow-up of prosthetic valve patients and to support the development of strategies for preventing this complication.

## 2. Materials and Methods

### 2.1. Study Design and Patient Selection

This study was designed as a retrospective, single-center, observational analysis. Clinical, demographic, and hemodynamic data from patients who underwent surgical intervention for stuck valve pathology between 2019 and 2024 at the Cardiovascular Surgery Clinic of Ankara Bilkent City Hospital were retrospectively reviewed. Ethical approval for the study was obtained from the Ankara Bilkent City Hospital Scientific and Ethics Review Committee for Medical Research (Approval No: TABED 1-24-619). The study was conducted in accordance with the Declaration of Helsinki, ensuring patient confidentiality and anonymizing all data.

Patients included in this study were adults aged 18 years and older who underwent surgical intervention and had a confirmed diagnosis of impinged valve. To ensure comprehensive data collection, only patients with complete medical records were included. Patients were excluded if they had incomplete medical records or presented with preoperative conditions such as active infections, septic symptoms, or other conditions that could confound valve dysfunction assessment.

Regarding thrombus formation, all patients were evaluated using transthoracic echocardiography and fluoroscopy to detect the presence of thrombus. Despite the careful selection of patients with significant valve dysfunction, thrombus formation was not observed in any patients during the course of the study. This may be attributed to the advanced imaging techniques employed, which effectively ruled out thrombus as a contributing factor to valve obstruction. Therefore, the study focused on pannus-related valve dysfunction, with thrombus formation not being a significant concern in the patient cohort.

### 2.2. Data Collection

The data of the patients included in the study were obtained retrospectively through the hospital’s electronic medical record system. Data collected included demographic characteristics (age, gender), comorbidities (hypertension, diabetes, coronary artery disease, chronic obstructive pulmonary disease), time since the first valve surgery, valve type (metallic or bioprosthetic), diagnosis of impinged valve, and valve pathology (pannus or thrombus formation). In addition, hemodynamic parameters such as pre- and postoperative left ventricular ejection fraction and systolic pulmonary artery pressure were recorded.

### 2.3. Imaging Methods

**Figure 1:**
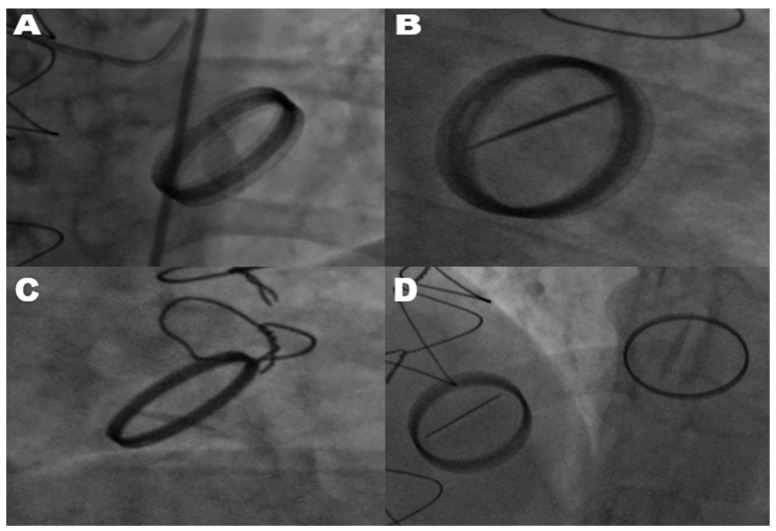
Fluoroscopic images of restricted leaflet movement in stuck prosthetic valves. This figure shows a fluoroscopic assessment of restricted leaflet movement in stuck metallic bileaflet mitral, aortic, and tricuspid valves, likely due to the mechanical obstruction caused by pannus. (**A**) Shows the mitral valve in a partially open position, with only one leaflet exhibiting movement, indicating a significant obstruction affecting valve function. (**B**) Depicts the mitral valve in an open position, with one leaflet remaining immobile, further demonstrating a mechanical limitation likely due to pannus. (**C**) Illustrates a stuck bileaflet metallic aortic valve, where the restricted leaflet movement suggests interference at the valve plane. (**D**) Displays a patient with previous metallic bileaflet mitral valve replacement and tricuspid valve replacement. In this view, only one leaflet of the stuck tricuspid valve is moving, highlighting that there is also an obstruction in the tricuspid valve. Additionally, the metallic bileaflet mitral valve is functioning normally, with both leaflets opening fully.

The diagnosis of mechanical prosthetic valve dysfunction, specifically a stuck valve, was confirmed through the combined use of transthoracic echocardiography and fluoroscopy. Transthoracic echocardiography was employed as the primary modality to evaluate leaflet mobility, detect the presence of thrombus or pannus, and assess associated hemodynamic alterations. Fluoroscopy complemented echocardiographic findings by directly visualizing the mechanical motion of valve leaflets. In cases where only one leaflet demonstrated motion, fluoroscopy reliably identified restricted leaflet mobility, confirming a stuck valve diagnosis. Echocardiography and fluoroscopy, when used in tandem, significantly improved the diagnostic accuracy of stuck valve identification and minimized the risk of misdiagnosis. This two-tiered assessment approach ensured the reliability of the diagnosis, enabling clinicians to proceed with surgical intervention despite the elevated operative risks associated with reoperation. All mechanical valves included in this study were bileaflet prosthetic valves, as verified through surgical and echocardiographic records. This uniformity in valve type minimized the variability stemming from differences in valve design, ensuring the study’s focus on pannus formation and hemodynamic gradients remained consistent. As shown in Figure 1, fluoroscopic imaging highlights the hallmark feature of limited leaflet motion in bileaflet metallic valves. This restriction in leaflet mobility significantly impairs hemodynamic function and was observed across various valve positions, including the mitral, aortic, and tricuspid valves.

### 2.4. Surgical Technique

**Figure 2:**
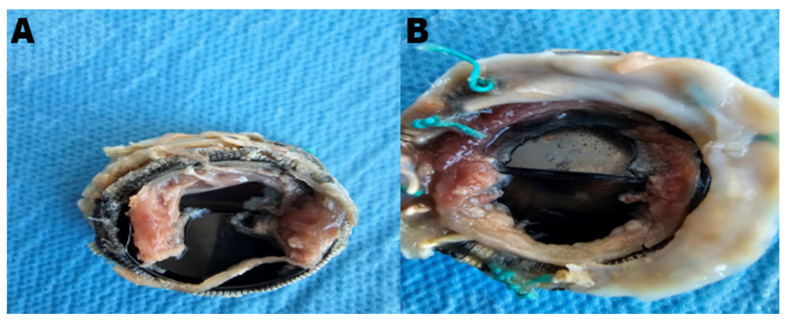
Key stages and pathologies of a stuck mitral bileaflet valve. In Figure 2, the key stages and pathologies associated with the stuck mitral bileaflet valve are displayed: (**A**) presents the excised valve, highlighting that pannus formation affected the valve’s functionality; (**B**) provides a close-up view of the valve with extensive pannus tissue encroaching on the leaflets, demonstrating the mechanical obstruction that evidently led to the valve’s stuck condition.

Patients with a confirmed diagnosis of a stuck valve underwent either pannus cleaning or valve replacement, depending on suitability. Pannus cleaning was performed in five suitable patients, while valve replacement was conducted in the remaining twenty-two patients. During surgery, the pathological findings, including pannus and thrombus formations on the excised valve, were thoroughly examined and documented. The decision between pannus cleaning and valve replacement was made based on the extent and localization of the pannus formation, as well as the overall structural integrity of the prosthetic valve. Pannus cleaning was performed in cases where the obstruction was localized and the valve structure was otherwise functional, while valve replacement was chosen for cases of extensive pannus formation or additional structural damage affecting valve performance. Pannus cleaning emerged as a valuable strategy in preserving the original valve, particularly in selected cases with localized pannus formation. As shown in Figure 2, the mechanical obstruction caused by pannus formation significantly impaired the movement of the valve leaflets, necessitating surgical intervention.

### 2.5. Pannus Cleaning Procedure

Pannus cleaning was performed during a standard cardiopulmonary bypass with systemic hypothermia (28–32 °C) and aortic cross-clamping. After achieving full cardioplegic arrest, the prosthetic valve was exposed via the corresponding chamber (left atrium for mitral valves, aortotomy for aortic valves, and right atrium for tricuspid valves). Careful inspection of the valve revealed the extent and localization of the pannus tissue. The fibrotic pannus, characterized by its firm and adhesive texture, was meticulously excised using fine surgical scissors and microsurgical forceps under direct visualization with high-resolution magnification.

The procedure involved careful dissection to avoid damaging the valve’s metallic framework and leaflets. Special attention was paid to preserving leaflet integrity while ensuring the complete removal of obstructive pannus tissue. In cases where pannus extended beneath the valve frame, a deeper dissection was performed, guided by intraoperative transesophageal echocardiography, to confirm the adequacy of pannus removal.

After pannus excision, the valve’s mobility was tested by manually cycling the leaflets to ensure functionality was restored. The surgical field was thoroughly irrigated with saline to remove any debris that might compromise valve performance postoperatively. Finally, the surgical site was carefully closed, and the patient was weaned off bypass under close hemodynamic monitoring to assess valve performance.

### 2.6. Statistical Analysis

All statistical analyses were conducted using IBM SPSS Statistics for Windows, Version 25.0 (IBM Corp., Armonk, NY, USA). Descriptive statistics were used to summarize demographic and clinical characteristics, with continuous variables presented as mean ± standard deviation and categorical variables as percentages. Comparisons between the mitral and aortic valve groups were assessed using chi-square tests for categorical variables and one-way ANOVA or independent sample *t*-tests for continuous variables. The tricuspid valve patient was not included in the comparisons due to the lack of statistical representation (*n* = 1). Where appropriate, post hoc comparisons were applied to evaluate the differences between specific groups. To evaluate the impact of pannus formation on critical parameters, such as time since the initial surgery and valve gradients, independent *t*-tests were performed. Additionally, preoperative and postoperative changes in ECG rhythm were analyzed using chi-square tests, while differences in preoperative and postoperative LVEF values were assessed with paired *t*-tests across the intervention groups (pannus cleaning, metallic valve replacement, and biological valve replacement). A *p*-value of <0.05 was considered statistically significant for all analyses.

## 3. Results

The demographic and clinical features of the 27 patients undergoing mitral, aortic, and tricuspid valve surgeries are summarized in Table 1. The mean age across all patients was similar, with mitral valve patients averaging 52.4 years and aortic valve patients 51.7 years. Only one patient underwent tricuspid valve replacement, who was female, while the other groups had a female predominance of around 60%. All explored valves in this study were bileaflet mechanical valves.

Hypertension was prevalent across all valve types, observed in 88.9% of mitral and 86.7% of aortic valve patients and in the one tricuspid valve patient. Diabetes mellitus was most common in the mitral valve group (27.8%) and was less prevalent in the aortic valve group (13.3%). Coronary artery disease was present in 33.3% of mitral valve patients but less common in aortic patients (6.7%) and absent in the tricuspid valve patient. Chronic obstructive pulmonary disease (COPD) was noted in 33.3% of mitral valve patients and 6.7% of aortic valve patients.

The time since initial surgery was also evaluated, with mitral valve patients showing a mean duration of 10.8 years, aortic valve patients a duration of 9.6 years, and the tricuspid valve patient a duration of 8.2 years. Statistical analysis revealed no significant differences among the valve groups in terms of demographic or clinical variables, including time since the initial surgery (*p*-values > 0.05).

Table 2 outlines the surgical outcomes and postoperative recovery data. The mean intensive care unit (ICU) stay was slightly longer for aortic valve patients (3.2 days) compared to mitral patients (2.2 days), but this difference was not statistically significant (*p* = 0.36). Similarly, the mean ward stay did not differ significantly among the groups (*p* = 0.63). No postoperative morbidities were reported in any of the groups. Mortality rates were 11.1% for mitral valve patients and 6.7% for aortic valve patients, with no mortality in the tricuspid valve patient; however, these differences were not statistically significant (*p* = 1.0). Among the three patients who experienced mortality, one belonged to the pannus removal group, while the other two were in the valve replacement group.

Table 3 compares critical variables between the pannus cleaning and valve replacement groups in patients with valve dysfunction. The variables analyzed include time since the initial surgery, maximum gradients, and mean gradients.

There was no significant difference in total time between the pannus cleaning group and the valve replacement group (*p* = 0.67). However, in mitral valve patients, the time since the initial surgery was longer in the valve replacement group (*p* = 0.02). For aortic valve patients, no significant difference was found (*p* = 0.24).

In the aortic valve group, no significant difference was observed between maximum and mean gradients (*p* = 0.12 and *p* = 0.08, respectively). In the mitral valve group, the maximum gradient was significantly higher in the pannus cleaning group compared to the valve replacement group (*p* = 0.03). However, no significant difference was found in mean gradients between the groups (*p* = 0.65).

Table 4 summarizes the hemodynamic and procedural outcomes of patients undergoing intervention for valve dysfunction, reflecting preoperative and postoperative rhythm distributions as well as changes in left ventricular ejection fraction (LVEF). All patients initially had metallic valves.

In the pannus cleaning group (*n* = 5), preoperative electrocardiographic (ECG) analysis revealed that 60% of patients (3/5) had a normal sinus rhythm (NSR), while 40% (2/5) presented with atrial fibrillation (AF). In the valve replacement group (*n* = 22), 82% of patients (18/22) exhibited an NSR, whereas 18% (4/22) had AF. These differences in preoperative rhythm distribution were not statistically significant (*p* = 0.675), indicating comparable baseline rhythm profiles across groups.

Postoperative ECG data showed distinct outcomes. In the pannus cleaning group, of the three patients with preoperative NSR, one developed complete atrioventricular (AV) block, while the remaining two maintained NSR. Among the two patients with preoperative AF, both remained in AF after surgery. In contrast, in the valve replacement group, 73% of patients (16/22) maintained NSR postoperatively, while 27% (6/22) were in AF, including two patients who transitioned from preoperative NSR to postoperative AF.

Regarding ventricular function, preoperative LVEF values were 45% ± 6% in the pannus cleaning group, 42% ± 5% in the metallic valve replacement group, and 40% ± 4% in the biological valve replacement group. Postoperatively, LVEF values increased to 52% ± 5%, 48% ± 6%, and 47% ± 5%, respectively, indicating an improvement in ventricular function after intervention. The preoperative-to-postoperative improvements in LVEF were statistically significant in all groups (*p* < 0.001).

## 4. Discussion

This study provides valuable insights into the multifactorial challenges associated with stuck prosthetic valves, highlighting the complex interactions between pannus formation, elevated gradient values, and rhythm disturbances. While our findings support those of previous studies, they are also important to consider when refining surgical and clinical management in this high-risk patient group. However, further research is needed to confirm these insights and establish more precise strategies for optimizing patient care in such cases.

The observed strong association between elevated gradient values and valve obstruction, particularly in mitral valves, emphasizes the localized mechanical impact of pannus formation. In our study, patients in the valve replacement group for mitral valves exhibited significantly higher maximum gradients (*p* = 0.03), suggesting that pannus formation contributes directly to leaflet restriction and increased transvalvular pressure [10]. This observation aligns with the findings of Faraj et al. (2023) [11], who reported that the elevated gradients in mitral valves are critical indicators of hemodynamic deterioration, including pulmonary congestion and edema. Consequently, comparisons of gradient values across valve types should be interpreted cautiously, considering the specific hemodynamic context of each valve. These findings are consistent with studies suggesting that gradient measurements alone may not be universally reliable diagnostic markers, particularly in aortic valves, where mechanical dysfunction may be influenced by factors other than gradients (e.g., calcification or thrombus burden). This underlines the importance of developing valve-specific diagnostic thresholds to guide clinical decision-making and intervention strategies. Furthermore, due to the higher blood flow velocity in the aortic valve localization compared to the mitral valve, thrombus or pannus formation is observed less frequently in the aortic valve position despite the higher gradient values [12,13].

Anticoagulation therapy, despite its well-established efficacy in preventing thrombus formation, was insufficient in mitigating pannus-related obstruction in our patient cohort. This supports the conclusions of Olson et al. (2019), who highlighted the ineffectiveness of pharmacological therapies in addressing pannus formation due to its fibrotic and inflammatory nature. The inadequacy of anticoagulation therapy reinforces the need for regular imaging follow-up, particularly with advanced modalities such as transthoracic echocardiography and fluoroscopy [14]. In our study, these imaging techniques proved invaluable in identifying early mechanical obstructions, allowing for timely intervention before significant clinical deterioration occurs. Comprehensive follow-up protocols integrating these tools are thus essential to improve long-term outcomes in patients with prosthetic valves.

Surgical intervention is essential for the management of stuck prosthetic valves, with pannus cleaning and valve replacement offering distinct advantages and limitations based on the extent of the valve obstruction and structural damage. The decision to perform pannus cleaning or valve replacement was primarily guided by the extent and location of pannus formation. In cases where the obstruction was localized and the valve structure remained intact, pannus cleaning was prioritized to preserve the original valve and reduce procedural risks. Conversely, valve replacement was chosen for cases involving extensive pannus formation or structural damage to the valve. This approach allowed for the comprehensive removal of obstructive tissue, particularly beneath the metallic frame, which is often challenging when using pannus cleaning alone. All mechanical valves in this study were bileaflet prosthetic valves, ensuring uniformity in design and reducing confounding factors related to valve-specific variability. Pannus cleaning was prioritized in cases of localized obstruction with intact valve structures, while valve replacement was chosen for extensive pannus formation or structural damage, ensuring the comprehensive restoration of valve function. These findings underscore the importance of tailoring surgical strategies to individual patient needs. In aortic valves, valve replacement requires extensive tissue excision, which increases the procedural risks, including the risk of adhesion. However, this approach allows for thorough pannus removal, potentially reducing recurrence rates and improving outcomes. Pannus cleaning emerged as a valuable strategy to preserve the original valve, particularly in selected cases with localized pannus formation [15,16]. All mechanical valves included in this study were bileaflet prosthetic valves. These considerations should guide surgical decision-making, balancing procedural complexity with long-term outcomes. However, the absence of other valve types in the study limits the generalizability of the findings to such designs. Consistent with previous studies, pannus cleaning, in selected cases, reduces surgical morbidity compared to valve replacement [15,17]. In our cohort, one patient in the pannus cleaning group developed complete atrioventricular block postoperatively, and atrial fibrillation persisted in others. While this complication may arise due to the proximity of the pannus to the conduction system, its direct association with the surgical approach remains uncertain. Re-replacement, which involves more extensive tissue excision, was not associated with a higher incidence of AV block in our cohort. Further studies are needed to delineate the specific risk factors contributing to AV block in different surgical strategies. While pannus cleaning is less invasive, it still carries procedural risks, such as the possibility of atrioventricular block, highlighting the importance of precise preoperative assessment and intraoperative techniques. Similarly, valve replacement—particularly in aortic valves—may lead to procedural complications such as adhesion-related injuries, emphasizing the necessity of meticulous preoperative assessment and intraoperative precision in both approaches.

Valve replacement, particularly with biological valves, demonstrated superior rhythm stabilization in our study. While our findings suggest that biological valve replacement may promote rhythm stabilization, these observations are based on a very small sample size (*n* = 2). As such, any conclusions regarding the superiority of biological valves in this context should be interpreted with caution. Further studies with larger cohorts are needed to validate these findings. In our cohort, two patients under the age of 50 underwent biological valve replacement. The decision to use biological valves in these cases was guided by specific clinical factors, including contraindications to long-term anticoagulation and a history of atrial fibrillation. While the risk of early valve degeneration in younger patients is recognized, the anticipated benefits of reduced thromboembolic risk and rhythm stabilization outweighed these concerns in these particular cases. Nevertheless, long-term follow-up is necessary to assess the durability of these valves and their impact on patient outcomes. Patients undergoing biological valve replacement achieved 100% normal sinus rhythm (NSR) postoperatively, a finding consistent with that of Xiang et al. (2021), who reported similar benefits in patients undergoing biological valve replacement for advanced prosthetic valve disease [18]. Biological valves’ ability to reduce the burden of atrial fibrillation and promote rhythm stability makes them a compelling choice, particularly in patients with contraindications to anticoagulation or a history of infective endocarditis. In contrast, mechanical valve replacement was associated with a 27% incidence of postoperative atrial fibrillation, including cases transitioning from preoperative NSR. These findings, supported by Badhwar et al. (2012) [19], suggest that while mechanical valves offer durability, their use may necessitate a closer rhythm monitoring and tailored postoperative care to address potential arrhythmias. Our results underscore the importance of individualizing valve selection based on patient-specific factors, balancing mechanical durability with rhythm-related and hemodynamic considerations.

The significant improvements in LVEF observed across all intervention groups (*p* < 0.001) highlight the efficacy of surgical strategies in restoring ventricular function. Despite these positive outcomes, the mortality rate observed in our cohort underscores the challenges associated with managing complex prosthetic valve dysfunctions. Among the three patients who experienced mortality, two were in the valve replacement group and one was in the pannus cleaning group. These cases were characterized by severe preoperative conditions, including an advanced NYHA class (III-IV), reduced left ventricular ejection fraction (LVEF ≤ 35%), and, in one case, severe pulmonary hypertension. These findings emphasize the critical role of preoperative optimization in improving surgical outcomes and reducing mortality risk. Future studies should aim to stratify risk factors more comprehensively to enhance patient selection and perioperative management. This finding reinforces the critical role of timely surgical intervention in mitigating the detrimental cardiac effects of valve obstruction. By normalizing LVEF, these interventions address systemic hemodynamic dysfunction, ultimately improving patient outcomes and quality of life [20,21]. The consistency of LVEF improvements across both pannus cleaning and valve replacement groups also underscores the ability of these interventions to restore effective cardiac performance, irrespective of the approach. These findings resonate with the broader clinical evidence supporting the benefits of early surgical correction in managing prosthetic valve complications and preventing long-term ventricular remodeling. In addition to the observed improvements in LVEF, all patients demonstrated significant recovery in terms of postoperative valvular functional status, with clinical assessments indicating a return to a normal prosthesis function. This consistent improvement across intervention groups reflects the success of surgical approaches in alleviating prosthetic valve obstruction and restoring effective cardiac output, ultimately contributing to improved ventricular function.

The observed differences in post-surgical atrial fibrillation outcomes between the biological and mechanical valve replacement groups require further explanation. It is important to note that the pressure gradients were indeed lower in the biological valve group, which may contribute to the observed reduction in atrial fibrillation. However, this finding should be interpreted cautiously, as other factors, such as valve durability, thromboembolic risk, and the patient’s preoperative rhythm disturbances, may have played significant roles. Given the small sample size of biological valve cases, these observations are preliminary and need confirmation in larger, more diverse patient populations.

Our study’s observations contribute to a growing body of evidence emphasizing the need for a multidisciplinary approach to the management of stuck prosthetic valves. Integrating surgical expertise, advanced imaging, and patient-specific risk stratification into clinical workflows can improve outcomes and reduce the burden of valve-related complications [22]. Furthermore, our findings support ongoing efforts to refine diagnostic and therapeutic strategies, including the development of novel valve technologies and bioengineered solutions to address the limitations of the current approaches.

This study has several limitations that should be considered when interpreting the findings. First, the retrospective design introduces inherent biases, including potential inaccuracies in data collection and reliance on pre-existing medical records. The small sample size, particularly in the biological valve replacement group (*n* = 2) and pannus cleaning group (*n* = 5), limits the generalizability of the results and restricts the ability to perform a robust subgroup analyses. The single-center nature of the study may further reduce the applicability of the findings to diverse clinical settings, as institutional practices and surgical expertise can vary significantly.

Additionally, the lack of data on anticoagulation adherence and appropriateness restricts the ability to clinically distinguish pannus from thrombus formation. The potential role of thrombolysis as a first-line therapy in selected cases was not explored in this study, which represents another limitation. Furthermore, the absence of specific data on the opening and closing angles of mechanical valves limits the ability to comprehensively assess early leaflet mobility restrictions caused by pannus formation. The inclusion of such measurements in future studies could provide valuable insights into the dynamics of valve obstruction.

Lastly, the absence of detailed anticoagulation compliance data and long-term imaging follow-up restricts the evaluation of these factors’ impact on outcomes. Future research with a prospective, multi-center design and larger sample sizes is essential to validate these findings and provide a more comprehensive understanding of stuck valve management.

## 5. Conclusions

This study provides valuable insights into the multifactorial challenges associated with stuck prosthetic valves, highlighting the significant roles of pannus formation, elevated gradient values, and rhythm disturbances. The findings underscore the critical importance of early diagnosis and tailored treatment strategies to optimize patient outcomes. Pannus cleaning emerged as a potential alternative to valve replacement in selected cases, particularly when the obstruction is localized and the valve structure is intact. However, the procedure’s limitations, such as its impact on rhythm stabilization and the need for specialized surgical expertise, should be carefully considered.

Valve replacement, especially with biological valves, was associated with improved rhythm stabilization and reduced infection risk. However, the small sample size (*n* = 2) limits definitive conclusions about the broader applicability of this method. Further research with larger cohorts is needed to confirm these findings. Significant improvements in LVEF across all intervention groups demonstrate the effectiveness of surgical strategies in restoring ventricular function and alleviating the hemodynamic burden of valve obstruction.

These findings emphasize the importance of regular follow-up, advanced imaging techniques, and a multidisciplinary approach to ensure the optimal management of prosthetic valve complications. Future studies should involve larger, multi-center cohorts and explore emerging valve technologies, such as bioengineered prostheses, to overcome the current limitations and further improve patient outcomes while addressing the inflammatory processes associated with prosthetic valves.

## Figures and Tables

**Table 1:** Demographic and clinical characteristics of patients by valve type.

Variable	Mitral Valve (*n* = 13)	Aortic Valve (*n* = 13)	Tricuspid Valve (*n* = 1)	*p*-Value
Mean Age (years)	52.4 ± 8.7	51.7 ± 10.2	48.3	0.76
Female (%)	61.5%	61.5%	100%	0.95
Hypertension (%)	88.9%	86.7%	100%	0.85
Diabetes Mellitus (%)	27.8%	13.3%	0%	0.22
Coronary Artery Disease (%)	33.3%	6.7%	0%	0.08
COPD (%)	33.3%	6.7%	0%	0.08
Time Since Initial Surgery (years)	10.8 ± 4.5	9.6 ± 5.2	8.2	0.54

Tricuspid valve data (*n* = 1) are presented descriptively and were excluded from statistical comparisons. The *p*-values reflect comparisons between the mitral and aortic valve groups.

**Table 2:** Surgical outcomes and postoperative recovery by valve type.

Variable	Mitral Valve (*n* = 13)	Aortic Valve (*n* = 13)	Tricuspid Valve (*n* = 1)	*p*-Value
ICU Stay (days)	2.2 ± 1.1	3.2 ± 1.5	2.7	0.36
Ward Stay (days)	6.2 ± 2.0	6.8 ± 2.3	6.0	0.63
Morbidity (%)	0%	0%	0%	1.0
Mortality (%)	11.1%	6.7%	0%	1.0

Tricuspid valve data (*n* = 1) are presented descriptively and excluded from statistical analyses. The *p*-values represent comparisons between the mitral and aortic valve groups.

**Table 3:** Critical variables associated with valve dysfunction: comparison of pannus cleaning and valve replacement groups.

Variable	Pannus Cleanup Group (*n* = 5)	Valve Replacement Group (*n* = 22)	*p*-Value
**Time Since Initial Surgery (years)**			
Total	11.2 ± 8.9	11.9 ± 7.2	0.67
Aortic Valve	13.3 ± 8.8	11.3 ± 7.2	0.24
Mitral Valve	2.0 ± 1.4	13.3 ± 7.0	0.02
Tricuspid Valve	-	1.0 ± 0.0	-
**Max. and Mean Gradient (mmHg)**			
Aortic Valve	61.8 ± 30.0/32.8 ± 15.6	90.6 ± 27.2/55.1 ± 17.3	0.12/0.08
Mitral Valve	17.7 ± 32.3/10.5 ± 0.7	20.2 ± 7.2/12.1 ± 5.6	0.03/0.65
Tricuspid Valve	-	26.0 ± 0.0/17.0 ± 0.0	-

Comparisons were performed for mitral and aortic valves only. Tricuspid valve data were excluded due to the lack of samples in the pannus cleaning group, with only a single case in the valve replacement group. The *p*-values pertain to comparisons within the mitral and aortic valve groups.

**Table 4:** Hemodynamic and procedural outcomes for patients with stuck prosthetic valves.

Variable	Pannus Cleaning (*n* = 5)	Valve Replacement (*n* = 20)	Biological Valve Replacement (*n* = 2)	*p*-Value
Initial Valve Type	100% Metallic	100% Metallic	100% Metallic	-
Preoperative ECG Rhythm	60% NSR, 40% AF	82% NSR, 18% AF	100% NSR	0.675
Postoperative ECG Rhythm	40% NSR, 40% AF, 20% AV Block	73% NSR, 27% AF	100% NSR	-
Preoperative LVEF (Mean ± SD)	45 ± 6%	42 ± 5%	40%	<0.001
Postoperative LVEF (Mean ± SD)	52 ± 5%	48 ± 6%	47%	-
Postoperative Pressure Gradients (Max/Mean, mmHg)	29.2 ± 11.4/18.3 ± 8.2 (Aortic) 8.1 ± 3.2/5.2 ± 1.9 (Mitral)	28.1 ± 11.2/15.3 ± 8.5 (Aortic) 6.9 ± 2.8/4.8 ± 1.7 (Mitral)	5.2/3.0 (Tricuspid) 23/14 (Aortic)	-

NSR: normal sinus rhythm; LVEF: left ventricular ejection fraction; AF: atrial fibrillation; AV: atrioventricular block. *p*-values derived from chi-square test for ECG rhythm changes and *t*-tests for LVEF values. Comparisons were focused on pannus cleaning and metallic valve replacement groups. Biological valve replacement (*n* = 2) and tricuspid valve data (*n* = 1) are presented descriptively and excluded from statistical analyses. The *p*-values reflect comparisons between the pannus cleaning and metallic valve replacement groups for mitral and aortic valves.

## Data Availability

The raw data supporting the conclusions of this article will be made available by the authors on request.
